# Dark-matter matters: Discriminating subtle blood cancers using the darkest DNA

**DOI:** 10.1371/journal.pcbi.1007332

**Published:** 2019-08-30

**Authors:** Laxmi Parida, Claudia Haferlach, Kahn Rhrissorrakrai, Filippo Utro, Chaya Levovitz, Wolfgang Kern, Niroshan Nadarajah, Sven Twardziok, Stephan Hutter, Manja Meggendorfer, Wencke Walter, Constance Baer, Torsten Haferlach

**Affiliations:** 1 IBM Research, Yorktown Heights, New York, United States of America; 2 MLL Munich Leukemia Laboratory, Munich, Germany; University of Maryland Baltimore County, UNITED STATES

## Abstract

The confluence of deep sequencing and powerful machine learning is providing an unprecedented peek at the darkest of the dark genomic matter, the non-coding genomic regions lacking any functional annotation. While deep sequencing uncovers rare tumor variants, the heterogeneity of the disease confounds the best of machine learning (ML) algorithms. Here we set out to answer if the dark-matter of the genome encompass signals that can distinguish the fine subtypes of disease that are otherwise genomically indistinguishable. We introduce a novel stochastic regularization, *ReVeaL*, that empowers ML to discriminate subtle cancer subtypes even from the same ‘cell of origin’. Analogous to *heritability*, implicitly defined on whole genome, we use *predictability (F*_*1*_ *score)* definable on portions of the genome. In an effort to distinguish cancer subtypes using dark-matter DNA, we applied *ReVeaL* to a new WGS dataset from 727 patient samples with seven forms of hematological cancers and assessed the predictivity over several genomic regions including genic, non-dark, non-coding, non-genic, and dark. ReVeaL enabled improved discrimination of cancer subtypes for all segments of the genome. The non-genic, non-coding and dark-matter had the highest F_1_ scores, with dark-matter having the highest level of predictability. Based on ReVeaL’s predictability of different genomic regions, dark-matter contains enough signal to significantly discriminate fine subtypes of disease. Hence, the agglomeration of rare variants, even in the hitherto unannotated and ill-understood regions of the genome, may play a substantial role in the disease etiology and deserve much more attention.

## Author summary

Many subtypes of cancer are unable to be distinguished based on their genomic profiles. With the ever-increasing use of sequencing, we now have the ability to look deeper into the genome and pick up on hidden signals in areas typically considered irrelevant to disease. To overcome the issue of rare variants and the vast amount of heterogeneity found in these non-coding sectors, we introduce a new algorithm capable of correcting for both challenges, *ReVeaL*. Using this approach, we are able to demonstrate that the non-coding regions of the genome have more signal for distinguishing subtle subtypes of disease compared to all the coding regions. Specifically, we show that the darkest unexplored genomic regions, the non-coding genome with no functional annotation whatsoever in the literature, have the strongest signal. Thus dark-matter does indeed matter and should not be ignored but rather considered for the continued pressing task of finding biomarkers of disease to adequately treat our patients.

## Introduction

Since the completion of the Human Genome Project, progress has been made in understanding the genome, particularly in diseases of the genome such as cancer. However, large gaps continue to exist in our knowledge of mutational (genomic) markers *vis-à-vis* subtle disease subtypes. Equipped with ultra-deep whole genome sequencing (WGS) capabilities that dig out ever more rare variants and current machine learning (ML) capabilities with the potential to process large amounts of data undeterred by noise at various scales, we focus here on blood cancer. Does the genome encompass signals that can differentiate the fine subtypes of the disease?

The whole genome (WG) has not been uniformly probed in oncology research. The primary focus has been on coding genes; the assumed instigators of cancer. Whole exome sequencing’s (WES) intrinsic focus on coding DNA, called the exonic, has naturally reinforced the centrality of coding genes as “cancer drivers” by exclusively discovering coding alterations associated to disease etiology. Although the exonic only covers only about 3% of the WG, the proteins encoded here have become the markers used for diagnosis, prognosis, and treatment plan design. Classical oncogenetics believe passenger mutations accompany driver mutations throughout the genome but are inconsequential to tumorigenesis [1]. Recently this model has been redefined with the suggestion that these passenger mutations, whether in coding or non-coding DNA, might actually have a role in cancer progression [2]. The aggregate effect of multiple weak passenger mutations may have a strong influence on tumorigenesis.

With the increasing use of WGS, genomic variation is gradually being revealed in non-coding DNA, the historically ignored region of the genome [3]. Researchers have noticed portions of the non-coding region are actually ultra-conserved, suggesting its vitality to life [4]. Regulation of transcription and translation of the DNA coding regions occurs within the non-coding and are potentially missed by WES. Annotated portions of the non-genic region contain regulatory elements such as promoters, enhancers, silencers, histone modifications, and ncRNA [5], accounting for about 7.3% of the WG. Research has begun to suggest that the unannotated non-genic regions, the dark-matter DNA occupying 52.86% of the WG, may have functional impact and the passenger mutations there-in may influence tumorigenesis [6]. Evidence for the presence of disease-associated signal in the dark-matter started surfacing in the literature, including its influence in brain development, bone metabolism, heart function, and a myriad of cancers [7–13].

Recently, broad cancer types have been successfully classified using epigenetic features alone. One suggested explanation is that distinctive epigenomic patterns are derived from the cells of origin rather than from the carcinogenic state and thus can be used for cancer type prediction [14, 15]. However, this theory does not address whether cancers from identical cells of origins can be separated using genomic markers. The question remains whether a cancer from the same few cells of origin has distinctive genomic signals capable of separating tightly related subtypes. Could those signals reside within the dark-matter? We set out to answer these questions using blood cancer patients, where the origin of the disease is in a few cells all developing from hematopoietic stem cells, yet the phenotype displays multiple subtypes of blood cancer.

## Results

**Fig 1:**
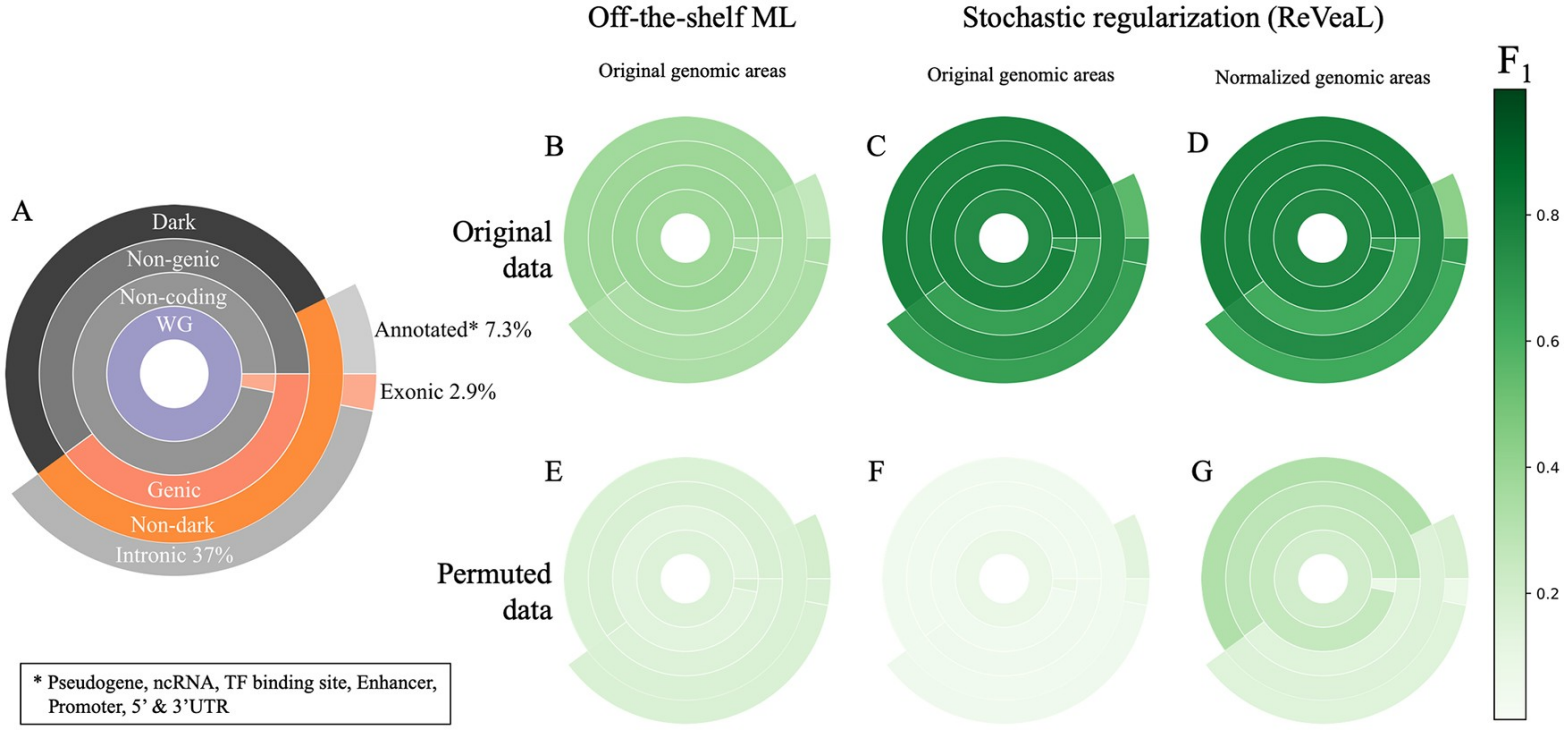
Partitions of genomic regions based on Ensembl annotation and their predictability of blood cancer WGS data. **(A)** The partition of the genomic region. **(B-G)** Median F_1_ values for the respective regions and their permuted controls when using off-the-shelf ML (B and E), ReVeaL on the original genomic areas (C and F) and ReVeaL on genomic areas normalized by length (D and G). See S2 Table for the F_1_ values.

In this manuscript we use predictability as an analogous concept of *heritability of traits* and take it to next level of inquiry by looking at segments of the genome and not just the whole [16]. We utilize the F_1_ measure, which is the (harmonic) mean of precision and recall, and like heritability satisfies 0 ≤ F_1_ ≤ 1, with similar interpretation. Specifically, we apply this concept to the analysis of five possible non-overlapping partitions of the WG taken from Ensembl annotation and shown as five concentric rings of genomic regions in Fig 1A. We applied this partitioning to a new 90X WGS dataset from 727 patient samples with seven forms of hematological cancers: acute myeloid leukemia (AML), B-cell acute lymphoblastic leukemia (B-ALL), chronic lymphoblastic leukemia (CLL), chronic myeloid leukemia (CML), chronic myelomonocytic leukemia (CMML), primary myelofibrosis (PMF), and myelodysplastic syndromes (MDS). When analyzing such large numbers of sequencing samples, it is common to mitigate the impact of intertumoral heterogeneity by considering the windows (segments) of bases instead of single bases [15].

We found the predictability (F_1_ score) of an array of ML and Artificial Intelligence (AI) methods on patient genomic data algorithms to be disappointingly poor when we considered multiple types of features including individual alleles, individual genes (utilizing mutational load, maximum allele frequency, binarized mutational state, or SnpEff predicted functional impact) (S5 Table), and windows of mutations from different genomic regions (exonic, annotated, genic, etc.) (Fig 1B). Off-the-shelf ML/AI methods obtained a median F_1_ score of only 0.34, 0.26 and 0.33 for the exonic, annotated, and genic genomic regions, respectively. Furthermore, the combination of all these regions, shown as non-dark, yielded an F_1_ score of 0.33. The poor performance of the off-the-shelf approach at either the allele, gene, or genomic region scale is not surprising as even the few genetic biomarkers incorporated into the WHO classification for hematological cancer [17] are largely non-exclusive between disease subtypes and exist at relatively low percentages (with the exception of the BCR-ABL fusion in CML).

The failure to reproduce expected predictability results on the exonic, genic, and annotated regions pointed us in two directions. One was to fortify the ML methods to address any possible weakness, and the second was to probe deeper into the typically ignored regions, i.e. the dark sector. We postulated that the heterogeneity in the population is perhaps confusing even for powerful ML/AI methodologies. Further, deep sequencing unearths unknown and unstudied rare variants that may collectively inform the ML/AI algorithms. To address these factors, we used a novel stochastic regularization that goes further in mitigating intertumoral heterogeneity than windows of genomic segments in an algorithm named *ReVeaL*. This approach is based on resampling and constructs ‘s*hingles’* that can be considered analogous to windows of earlier methods. Yet ReVeaL goes further by employing a stochastic regularization that transforms the raw mutation data into distributions per shingle that is then used by the learning algorithms to build models (S1 Fig). The reader is referred to the Material and Methods Section for further details.

Using ReVeaL we obtain F_1_ scores of 0.69, 0.56, 0.67 and 0.74 for the exonic, annotated, genic and non-dark regions, respectively (Fig 1C), which is better than off-the-shelf ML/AI methods. We also observed the L1 distance to scores from the permutation tests is much greater in ReVeaL than the off-the-shelf ML/AI methods. The respective number of features when looking over windows of the non-aggregated patient samples was comparable to the number of shingles, and the gene level feature space was comparable in size to the genic and exonic genomic segment shingles. Surprisingly, the non-genic, non-coding, and dark-matter show a ReVeaL F_1_ score of 0.77, 0.75, and 0.78, respectively, which is better than the non-dark sector of the genome. To rule out whether different sizes of genomic sectors are possibly affecting the F_1_ scores, we normalize the sector sizes and reproduce similar F_1_ scores (Fig 1D), concluding that ReVeaL’s performance is independent of the sector size. All the methods are permutation-tested and repeated 10 times to assess variability (details in Supplement).

All three modes (off-the-shelf AI/ML, ReVeaL, ReVeaL on normalized lengths) of analysis used 10 Monte Carlo splits with 75% training and 25% test, although similar results were observed on a wider range of training and test splits. The predictability score falls dramatically when the phenotype labels are randomly permuted (Fig 1E–1G) and the L1 distance between the original and permuted data is much higher with ReVeaL than the others (for example, 0.74 vs 0.21 in the dark sector). We further validated ReVeaL’s ability to discriminate diseases using broad cancer types from PCAWG [18] (S1 File).

The ReVeaL F_1_ score of the non-coding sector (0.75) is higher than that of the coding (0.69). The intronic and annotated sectors of the non-coding contain a large number of regulatory elements that may contribute to the disease separation. Yet, the F_1_ score of the dark sector (0.78) is higher than all non-coding (0.75), suggesting dark alone accounts for most, if not all, of the performance observed in the entire non-coding region. The algorithm’s ability to discriminate between disease subtypes using only information from the dark region, without any consideration of large structural variants or known biomarkers, confirms the existence of disease-specific information present there. We observed that 76% of variants in the dark are rare, with an allele frequency < 1%. The high prevalence of rare variants implies that most alterations are not consistently represented in the disease populations. ReVeaL manages this heterogeneity more effectively through its stochastic regularization by extracting the discriminating signal for a higher F_1_ score. We postulate that the agglomeration of some rare variants, even in the heretofore un-annotated and ill-understood dark sectors of the genome, play a role in the disease etiology or progression and can be harnessed to differentiate cancer subtypes even within the same cell of origin.

**Fig 2:**
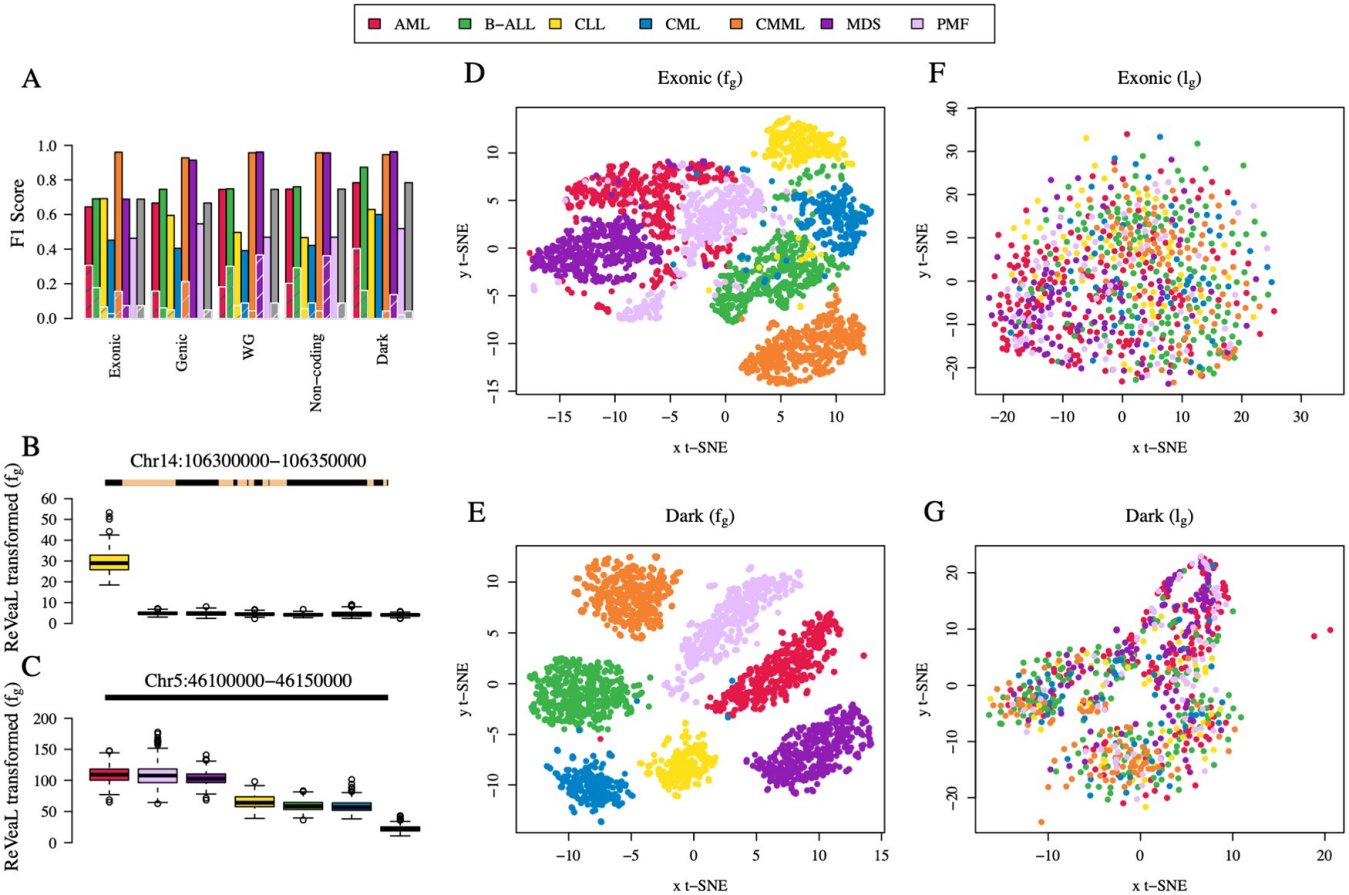
Disease-by-disease ReVeaL Analysis. **(A)** F_1_ scores for genomic sectors for each disease are averaged over all 10 replicate analyses per chromosome and the maximum F_1_ score is reported for that disease. ReVeaL scores on disease-label permutations are shown in overlaid hatched bars. The gray bar represents the mean over all diseases. **(B-C)** Boxplot of *f*_*g*_, shingle values representing the four moments of the distribution, of samples per disease and diseases ordered by decreasing median *f*_*g*_ for the top 2 ReVeaL features. The line above each boxplot represents the shingle, the yellow interval representing the portion of the segment that is masked. **(D-G)** t-SNE visualization (perplexity = 40, iterations = 300) using the top 50 shingle *f*_*g*_ values (B and C) and mutational load *l*_*g*_, number of mutations for a given window in the genomic region for a given patient, (D and E), respectively, in exonic and dark sectors.

We further analyzed the top 50 dark sector features across diseases. The t-Distributed Stochastic Neighbor Embedding (t-SNE—a non-linear dimensional reduction technique suitable for high-dimensional data) [19] visualization of the top features shows a clear separation of the different disease subtypes in the fifty-dimensional space using the dark sector shingle *f*_*g*_ values, representing the four moments of the distribution (Fig 2E). This is in stark contrast to the top 50 features of the coding/exonic sector (Fig 2D). Furthermore, the mutational load *l*_*g*_, number of mutations within the given windows, alone in the respective regions is not discriminative (Fig 2F and 2G). As an example, the top ranked feature that was consistently selected across all folds of the training and test is the genomic window *chr14*:*106300000–106350000* (Fig 2B). This region contains the Ig-epsilon locus, however, all mutations contained within annotated portions of this window were removed (or masked) leaving only alterations in the dark-matter region. While only considering those dark-matter alterations that surrond the Ig-epsilon locus, ReVeaL’s ablility to detect potentially functionally relevant unannotated regions was validated. The high *f*_*g*_ in CLL with respect to the other subtypes suggests a potential functional association between this dark feature and CLL. Another top ranked genomic window *chr5*:*46100000–46150000* contains no annotated/genic material and is therefore unmasked (Fig 2C). This window differentiates groups of diseases with AML, MDS, and PMF showing similar distributions from the group defined by B-ALL, CLL, and CML. CMML is an outlier disease indicating relative depletion of mutations in this region. Furthermore, there is no consistent mutational load (*l*_*g*_) ordering-by-disease across features as has been noted for other diseases [20].

## Discussion

In this study we discover how cancer subtypes sharing the ‘cell of origin’ can be classified using only their dark-matter signatures. This is the first study to successfully separate seven subtypes of leukemia using only dark-matter genomic regions. We use the touchstone of ML to examine the disease-*predictability* of the different genomic sectors. ML is robust and resilient to the presence of random noise. For instance, whether considering germline mutations, quality-controlled markers, or functionality of the alteration, we observe that the overall F_1_ is not affected in a significant manner, though they may help in reducing the problem size. Comparing the ReVeaL F_1_ across the blood cancer subtypes, we note that CLL is clearly discriminated from others only on chromosome 14 (S6 Fig), and CML has the lowest F_1_ in most genomic regions (Fig 2A). The latter is to be expected as the algorithms do not consider structural variants or known biomarkers, such as the BCR-ABL fusion that marks the CML disease class. Notably, CMML F_1_ is high for both genic and non-coding regions. For six of the seven diseases, the F_1_ performance remains similar or improves slightly when the non-coding is combined with the coding region, thus establishing the non-coding region is sufficient for distinguishing most leukemia subtypes.

While the discovery of discriminative signals in the dark region is novel and notable for its performance alone, there were several obstacles to this study concerning the availability of normal and negative controls. There were no matched normal tissues for the disease samples when calling variants and instead a normal population was drawn from individuals from a distinct genetic ancestry. Additionally, the negative controls used by ReVeaL were a permutation of the positive dataset rather than a set of data of normal patients stemming from the limited number of normal samples collected in a comparable fashion and sequenced to a similar depth. Given these limitations and the performance of ReVeaL against the permuted controls, the signal identified in the non-coding and dark sector remains important.

Discriminative features in the dark sector offers potential targets for functional studies to validate their role in their respective disease etiologies. In analyzing the top discriminating features found in the dark region, we were able to identify several that exhibited subtype specificity (Fig 2B and 2C). In addition, we found that the mutational load ordering-by-disease across features was not correlated with diseases as had been found in other cancer types. This supports the idea that the discriminative features in the dark sector are not simply mirroring global, non-coding mutational patterns traceable to the ‘cell of origin’ but are rather specific somatic alterations potentially influencing tumorigenesis.

Clearly epigenomic, transcriptomic, proteomic and all such -omics are powerful modalities that can only further our understanding of disease. Yet we find that the dark DNA alone carries subtype discriminating information. We introduce ReVeaL a well-designed algorithm that, in conjunction with rich data, is capable of hinting at the presence of these signals with irrefutable evidence (*ala* heritability of complex traits) even before actually probing that space. This speaks to the power of both deep sequencing technologies and algorithms. One of the key implications of our study is that there are potentially hitherto unknown modes of functionalities tucked away in the darkest of the unexplored genome.

## Material and methods

### Whole-genome data generation

A cohort of 727 patients was selected from patients for whom samples for routine leukemia diagnostics were sent to the Munich Leukemia Laboratory (Munich, Germany), representing seven different types of hematological malignancies: acute myeloid leukemia (AML, *n* = 185), B-cell acute lymphoblastic leukemia (B-ALL, *n* = 164), chronic lymphoblastic leukemia (CLL, *n* = 58), chronic myeloid leukemia (CML, *n* = 53), chronic myelomonocytic leukemia (CMML, *n* = 94), primary myelofibrosis (PMF, *n* = 71), and myelodysplastic syndromes (MDS, *n* = 102). All individuals involved in this study provided consent for publication. The study was approved by the Internal Review Board and adhered to the tenets of the Declaration of Helsinki. Genomic DNA was extracted from either peripheral blood or bone marrow samples using the DNA and Viral NA Large Volume Kit (Roche Diagnostics, Rotkreuz, Switzerland) and fragmented by ultrasonic shearing (Covaris, Woburn, MA), targeting an insert size of 350bp. Sequencing libraries were created using the TruSeq DNA PCR-Free Kit (Illumina, San Diego, CA) and 150bp paired-end sequencing was performed on Illumina Hiseq X and NovaSeq 6000 instruments to a mean coverage of 90x. Downstream processing of raw sequence data occurred within Illumina’s BaseSpace architecture (http://basespace.illumina.com). Reads were mapped to the GRCh37 reference genome using Isaac 3.16.02.19 [21] and somatic SNVs and short indels were called with Strelka 2.4.7 [22]. As matched-normal tissue was not available, a mixture of genomic DNA from gender-specific multiple anonymous donors (Promega, Fitchburg, WI) was used as an unmatched-normal for variant calling.

### Preprocessing genome data

WGS data was filtered for alterations that pass sequencing quality filters. Putative germline mutations were excluded by discarding all mutations present in ≥ 2% of the population according to gnomAD [12], as well as synonymous, *start_retained* and *stop_retained* mutations. To identify distinct genomic regions, we downloaded the Ensembl GRCh37 genome annotation GTF in July 2018 [23] and Ensembl Regulatory Build for genome version GRCh37 in Nov. 2016 [24]. The exonic sector consists of all regions indicated as *exon*. Genic is all *gene* annotated regions of ‘protein_coding’, ‘processed_transcript’ and ‘*_gene’ entries. Annotated non-coding regions are derived from all non-genic regions annotated as promoter, enhancer, transcription factor binding, and all other sites annotated in Ensembl GRCh37 (e.g. UTR, lincRNA, miRNA). All remaining non-genic mutations are considered dark.

### ReVeaL Analysis

ReVeaL, *Rare Variant Learning*, is a stochastic regularization-based learning algorithm. It partitions the genome into non-overlapping, possibly non-contiguous, windows (*w*) and then samples into possibly overlapping subsets, using subsampling with replacement (stochastic), giving units called *shingles* that are utilized by a statistical learning algorithm. Each shingle captures a distribution of the mutational load (the number of mutations in the window *w* of a given sample), and the first four moments are used as an approximation of the distribution.

We tested ReVeaL over a range of *w* values such as 5Kb, 25Kb and 50Kb, including instances where *w* represented cytoband-based or gene-based variable-sized windows. Patient samples were split for training/test. Although in data science literature 80/20 or even 90/10 split in quantitative genetics [25, 26] is recommended for training/test, we used a more conservative split of 75/25 here in this paper. Additionally, to have enough power in the data, we assume the availability of at least 50 samples for each disease subtype. We repeated the analysis 10 times with different random splits of the train and test subsets to determine variability and error of results along with features found consistently for each genomic sector.

To account for the relatively small size of the exonic and genic regions in comparison to the non-coding region, we tested ReVeaL with several different modes of partitioning the coding region, including partitioning at the gene boundaries, concatenating 10 sequential genes as a single partition, and concatenating all gene regions together and partitioning into fixed window sizes. We found there to be no strong difference between each of these partitioning strategies and used *w =* gene size for the coding regions. The procedure below provides a high-level description of the ReVeaL algorithm:

Let *Trn* be the training patient set and *Tst* be the test patient set of a fold.

1. Subsampling: Let *Sbsmp(S,k)* denote the procedure of sampling *k* (*k* = 35) random elements of *S* with replacement [25].
  For each fold, and each phenotype *p*,
  - Execute *y* times the stochastic procedure *Sbsmp (Trn,k*_*Trn*_*)* to get *y* training *samples*.
  - Execute *z* times the stochastic procedure *Sbsmp (Tst,k*_*Tst*_*)* to get *z* test *samples*.
  Each of the subset of marked patients is termed a *sample*. The *y + z* samples above is allocated the phenotype *p*.
2. Distribution: For each shingle (i.e. genomic window), we use the first four (central) moments as the approximation of its distribution [25]. In particular, for the t-SNE visualizations and a representative feature of the genomic window, we used the first moment of the shingle called *f*_*g*_.
3. Apply a ML/AI classification method on the data generated in step 2. We have used a collection of statistical learning algorithms on different subsets of the four moments.

### Negative controls

As a negative control, we produced a comparable data set to the input with no phenotype-differentiating signals. To achieve this, we permute the phenotype labels of patients before Step 1, i.e., passing the data through the training/test partition step. Similar to the standard process, we repeat this negative control analysis 10 times, each time the phenotype labels are permuted. We also provided an additional negative control of partitioning the genome without subsampling and aggregating samples where only *w* was constructed without forming the shingles and then provide as input to Step 3.

### Sector length normalization

Sector lengths were normalized by using a setting where each genomic sector covers only 2.9% of the WG—the size of the exonic region. The top discriminating shingles for all sectors, covering no more than 2.9% of WG, were selected.

### ML methods

The included ML methods fall into four broad categories: probabilistic (Naïve Bayes [25]), information theoretic (Random Forests [25]), linear/non-linear classification (Support Vector Machines (SVM) [25]), and neural networks (Multi-Layer Perceptron [25]). Each method was implemented with the R Statistical Language and in Python, primarily using the *scikit-learn* package (http://scikit-learn.org/). Parameters are noted in S6 Table.

### Scoring performance

Many different algorithms were tested to maximize the separation of the disease samples. An "algorithm" here is the specific combination of learning methodology and data staging. During the training and testing phase, for each fold, the learning methodology is trained on the training data and the resulting model is used to predict the labels of the test set. Performance is measured using the F_1_ score, which is the harmonic mean of recall and precision [14]. Scores are calculated per disease where positive, or ‘gold standard’, labels are the actual disease label of the sample.

### Feature selection

Feature selection was performed per split and used *LinearSVC* from the *scikit-learn* Python package. Genomic windows were treated as features using the shingle values *f*_*g*_ with an SVM cost penalty parameter *C* = 1.0. Features with non-zero support were selected for further investigation. In particular, the features were sorted according to their maximum support coefficient across all diseases. Finally, a consensus rank from across the multiple runs was computed, and the first 50 features were selected.

### Allele frequency (AF)

For each SNP in the data, the AF is computed as the number of patients having that mutation over the total number of patients.

## Supporting information

S1 File Method and result description of PCAWG analysis. (DOCX)

S1 Table Maximum F_1_ score and supporting parameters per disease for dark sector DNA using 50Kb windows. Unless otherwise noted, any tested value of the penalty *C* yielded equivalent results.
(XLSX)

S2 Table Median F_1_ values of genomic regions with different learning approaches in blood cancers. (XLSX)

S3 Table Median F_1_ values of genomic regions with different learning approaches in PCAWG. (XLSX)

S4 Table Position of masked biological entities for the top dark feature Chr14:106300000–106350000. (XLSX)

S5 Table Maximum F_1_ score respective data input from gene level analysis of mutation data where analyzed inputs included: Binary mutation state, mutational load, and SnpEff functional impact scores. (XLSX)

S6 Table ML algorithm parameters. (CSV)

S7 Table Performance metrics for all ML methods on ReVeaL input. (ZIP)

S1 Fig Flowchart of ReVeaL algorithm. ReVeaL computes the mutational load *l*_*g*_ from ‘Raw Input’ for a given disease, which is composed of all mutations for the given genomic sector of interest, e.g exonic or intronic, over a given genomic partition window size for each sample. Data is split to train and test sets, and within each subset, data is subsampled with replacement *k* times to compute aggregated shingles *f*_*g*_ over these subsampled data where a shingle is the distribution of *l*_*m*_ over the *k* samples as represented by the first four moments of the distribution. These shingles are used for classification and the process repeated *X* = 10 times. When computing the negative controls, the phenotype labels are permuted prior to the train/test split (diamond).
(TIF)

S2 Fig Partitions of genomic regions and their predictability of PCAWG data. A) The first center-most ring is a single partition (WG). The WG is partitioned into exonic and non-coding in the second; into genic and non-genic (the complement of genic) in the third; and into dark and non-dark in the fourth. In the final ring the non-dark is partitioned into exonic, intronic, and annotated. B-G) Mean F_1_ values for the respective regions and their permuted controls when using off-the-shelf ML (B and E), ReVeaL on the original genomic areas (C and F) and ReVeaL on genomic areas normalized by length (D and G). See S2 Table for the F_1_ values.
(TIF)

S3 Fig t-SNE of ReVeaL samples from blood cancers using top 50 features selected using a linear SVM on the distributions of the samples across all 10-splits in the respective regions. (TIF)

S4 Fig t-SNE of ReVeaL samples from PCAWG using top 50 features selected using a linear SVM on the distributions of the samples across all 10-splits in the respective regions. (TIF)

S5 Fig Disease-by-disease ReVeaL scores for all genomic sectors in PCAWG: Per-disease F_1_ scores are averaged over all 10-splits per chromosome and the maximum F_1_ score is reported for that disease. ReVeaL scores on disease-label permutations, used as negative controls, are shown in overlaid hatched bars.
(TIF)

S6 Fig Heatmap of SVM F_1_ scores per disease for the dark region partitioned into 50kbp windows. The cell represents the average F_1_ score over 10-splits for an SVM with a linear kernel and penalty C = 1.0 with a specific chromosome input indicated in the row name. Columns are organized by disease.
(TIF)

S7 Fig Disease-by-disease ReVeaL scores for all genomic sectors in blood cancers: Per-disease F_1_ scores are averaged over all 10-splits per chromosome and the maximum F_1_ score is reported for that disease. ReVeaL scores on disease-label permutations, used as negative controls, are shown in overlaid hatched bars.
(TIF)

S8 Fig Performance of ML methods on each genomic sector for ReVeaL. Each bar represents the mean over the set of maximum F_1_ scores achieved by any algorithm for a given disease.
(TIF)

S9 Fig Performance of ML methods on each genomic sector for non-ReVeaL data. Each bar represents the mean over the set of maximum F_1_ scores achieved by any algorithm for a given disease.
(TIF)
