# Supplementary material for: Dark-matter matters: Discriminating subtle blood cancers using the darkest DNA
Source: PLoS Comput Biol. 2019 Aug 30;15(8):e1007332. doi: 10.1371/journal.pcbi.1007332 (PMC6742441; doi:10.1371/journal.pcbi.1007332)
Supplement: S1 File — (DOCX) [file pcbi.1007332.s001.docx]

Supplementary

**Material and Methods**

*Whole-genome data PCAWG*

WGS data from the Pan-Cancer Analysis of Whole Genomes [18] was download from the ICGC (<https://dcc.icgc.org/pcawg>) in February 2018. A total of 2670 samples were downloaded, and precomputed somatic SNVs identified by Mutect and provided indels were used. Disease phenotypes were filtered for those with at least 50 samples, the minimum number for ReVeaL to be applied. Additionally, only chromosomes 1-22 were considered.

**Results**

*Discriminating major cancer types using PCAWG data*

Using the Pan-Cancer Analysis of Whole Genomes (PCAWG) dataset [18], ReVeaL was applied to WGS datasets across 15 diverse cancer disease types to determine if it is possible to separate these phenotypes based only on genomic information and not including established markers of disease. We only considered diseases with sufficient numbers of samples for ReVeaL to be appropriately applied, where that minimum number is 50. With a median F_1_ score of 1, ReVeaL analysis of the non-coding region was able to successfully distinguish all diseases (S4-5 Figure, S3 Table) using a simple SVM. Many diseases, including breast cancer (BRCA), esophageal adenocarcinoma (ESAD), and ovarian cancer (OV), had perfect separation. We also observed the performance of the exonic DNA was among the lowest performers for most diseases. Though the exonic region is also quite good at separating them with a mean F_1_ score of 0.96 and median of 0.98, a larger difference in performance was found for pediatric brain cancer (PBCA). Here both off-the-shelf ML/AI methods and ReVeaL perform well in almost all the genomic regions including the non-coding region (S4-5 Figure, S3 Table). However, ReVeaL’s performance also exceeds traditional mutational load analysis where only windowing on the genome is carried out, as seen in the increased F_1_ score for the exonic region moving from non-shingles to shingles. These results indicate that ReVeaL’s approach outperforms typical non-shingle analysis and the non-coding data is sufficient to discriminate between major cancer types and outperforms the exonic region alone.
