# Supplementary figures and images for: Dark-matter matters: Discriminating subtle blood cancers using the darkest DNA
Source: PLoS Comput Biol. 2019 Aug 30;15(8):e1007332. doi: 10.1371/journal.pcbi.1007332 (PMC6742441; doi:10.1371/journal.pcbi.1007332)

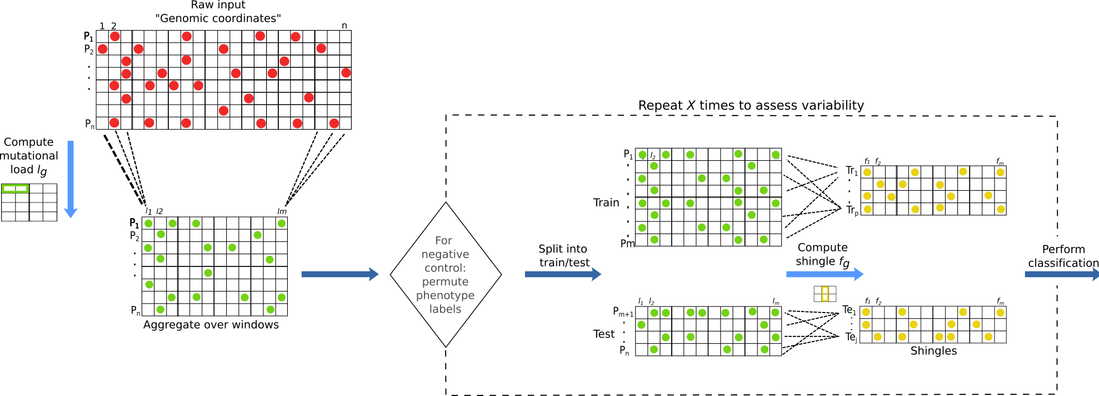

Supplement: S1 Fig — ReVeaL computes the mutational load lg from ‘Raw Input’ for a given disease, which is composed of all mutations for the given genomic sector of interest, e.g exonic or intronic, over a given genomic partition window size for each sample. Data is split to train and test sets, and within each subset, data is subsampled with replacement k times to compute aggregated shingles fg over these subsampled data where a shingle is the distribution of lm over the k samples as represented by the first four moments of the distribution. These shingles are used for classification and the process repeated X = 10 times. When computing the negative controls, the phenotype labels are permuted prior to the train/test split (diamond). (TIF) [file pcbi.1007332.s009.tif]

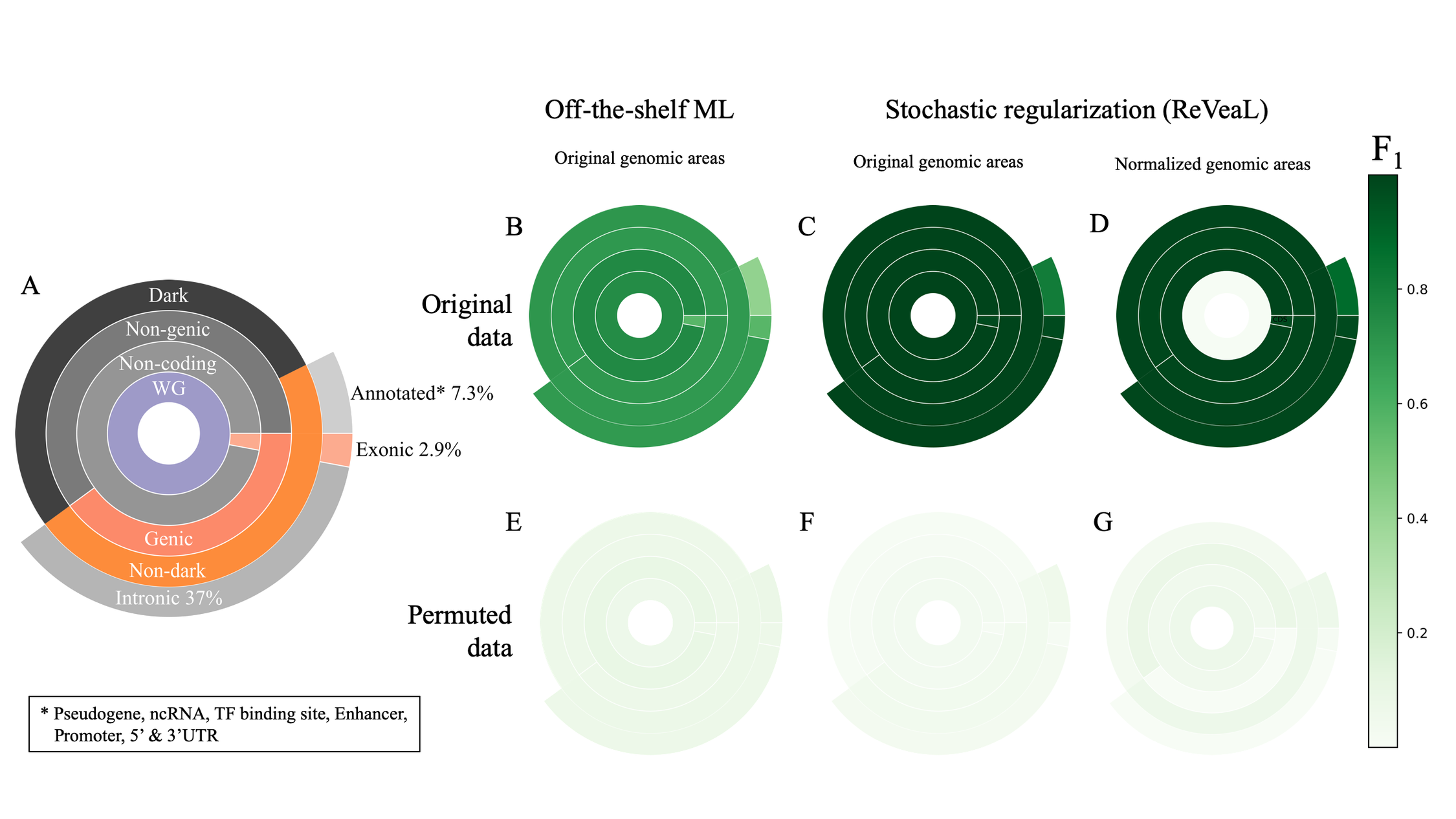

Supplement: S2 Fig — A) The first center-most ring is a single partition (WG). The WG is partitioned into exonic and non-coding in the second; into genic and non-genic (the complement of genic) in the third; and into dark and non-dark in the fourth. In the final ring the non-dark is partitioned into exonic, intronic, and annotated. B-G) Mean F1 values for the respective regions and their permuted controls when using off-the-shelf ML (B and E), ReVeaL on the original genomic areas (C and F) and ReVeaL on genomic areas normalized by length (D and G). See S2 Table for the F1 values. (TIF) [file pcbi.1007332.s010.tif]

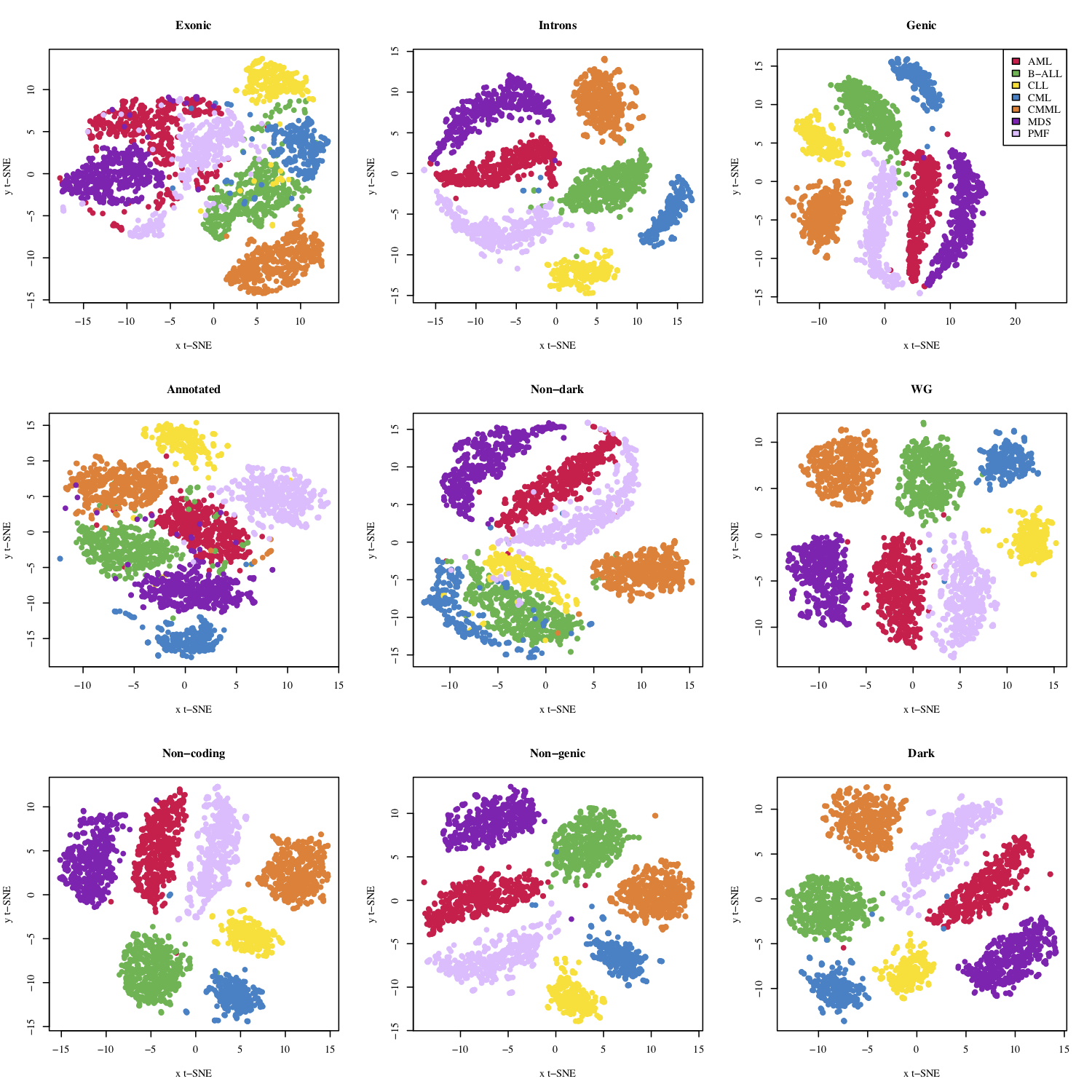

Supplement: S3 Fig — (TIF) [file pcbi.1007332.s011.tif]

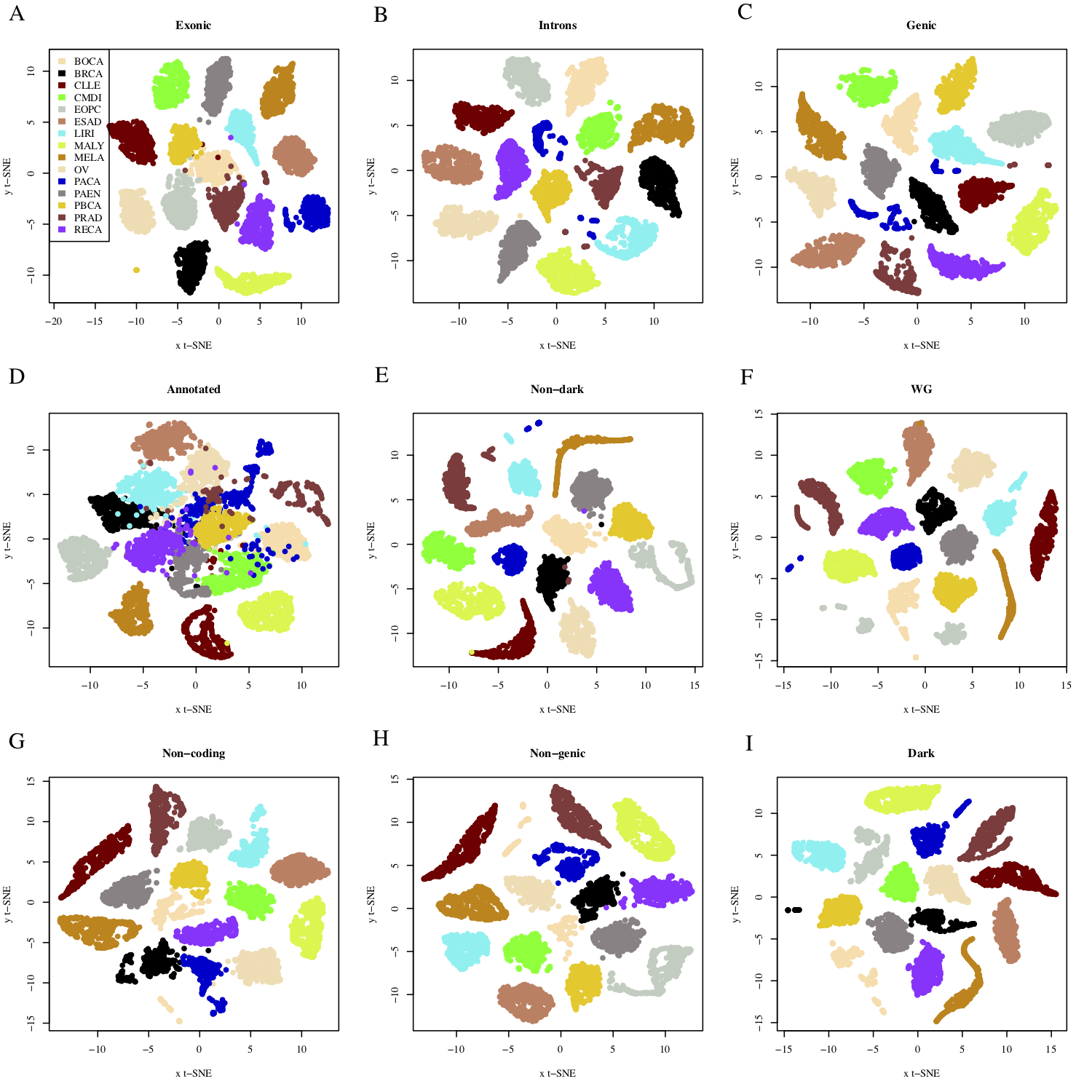

Supplement: S4 Fig — (TIF) [file pcbi.1007332.s012.tif]

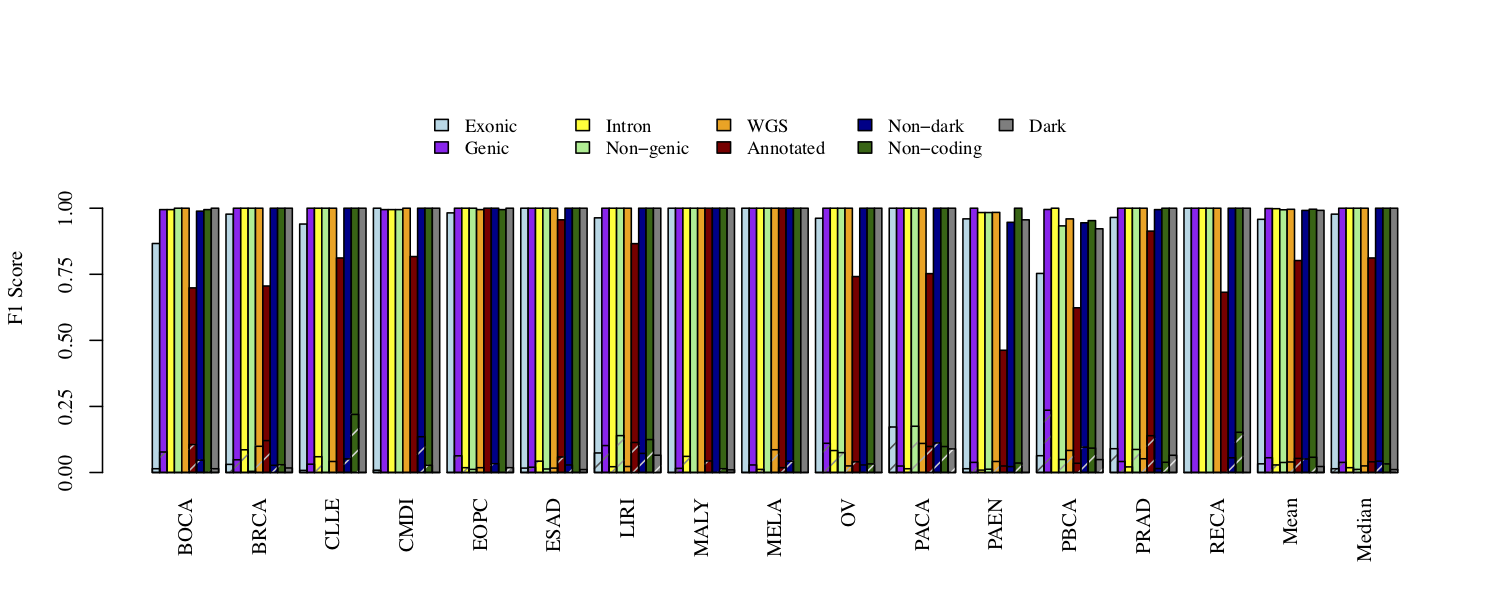

Supplement: S5 Fig — ReVeaL scores on disease-label permutations, used as negative controls, are shown in overlaid hatched bars. (TIF) [file pcbi.1007332.s013.tif]

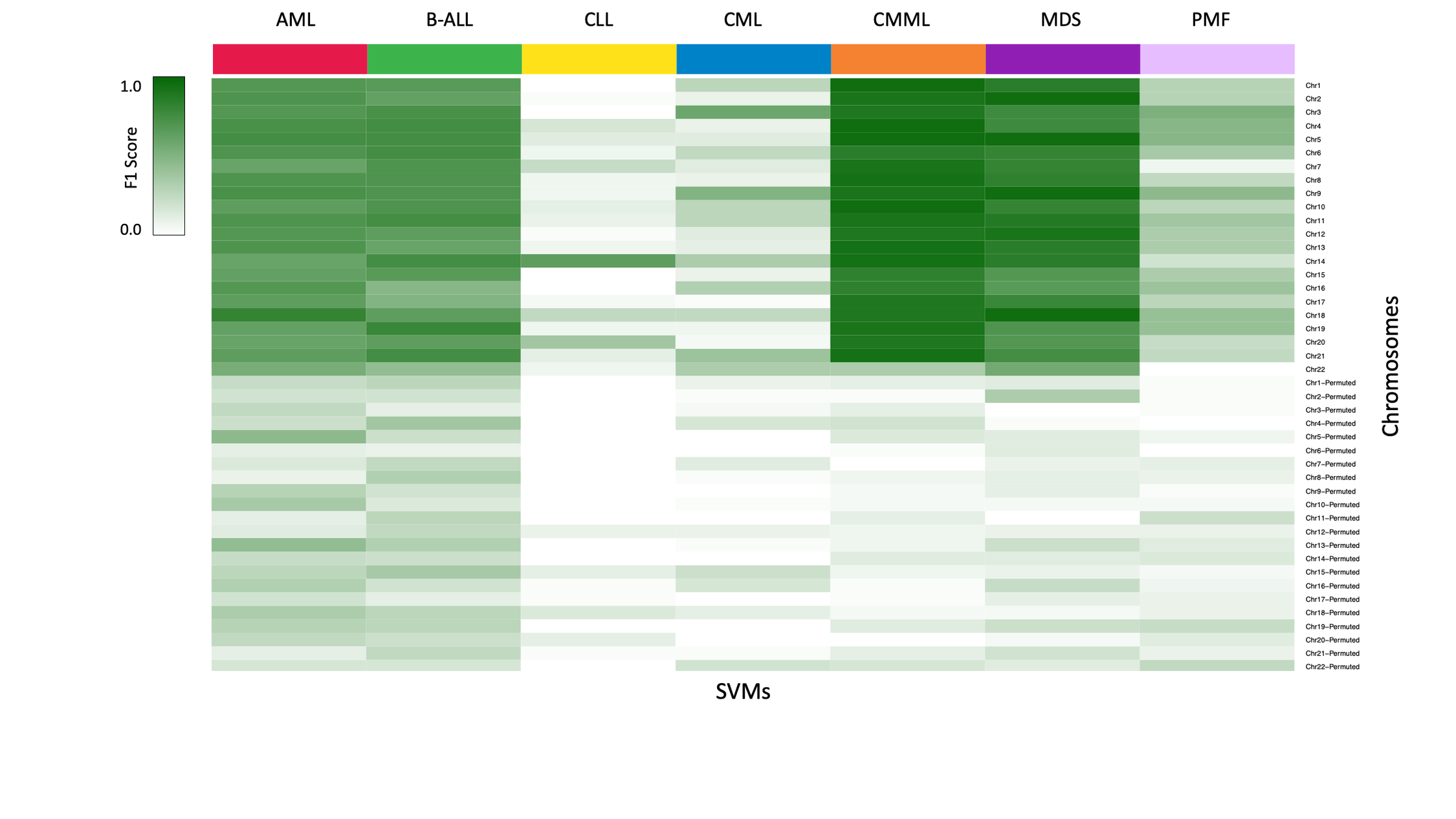

Supplement: S6 Fig — The cell represents the average F1 score over 10-splits for an SVM with a linear kernel and penalty C = 1.0 with a specific chromosome input indicated in the row name. Columns are organized by disease. (TIF) [file pcbi.1007332.s014.tif]

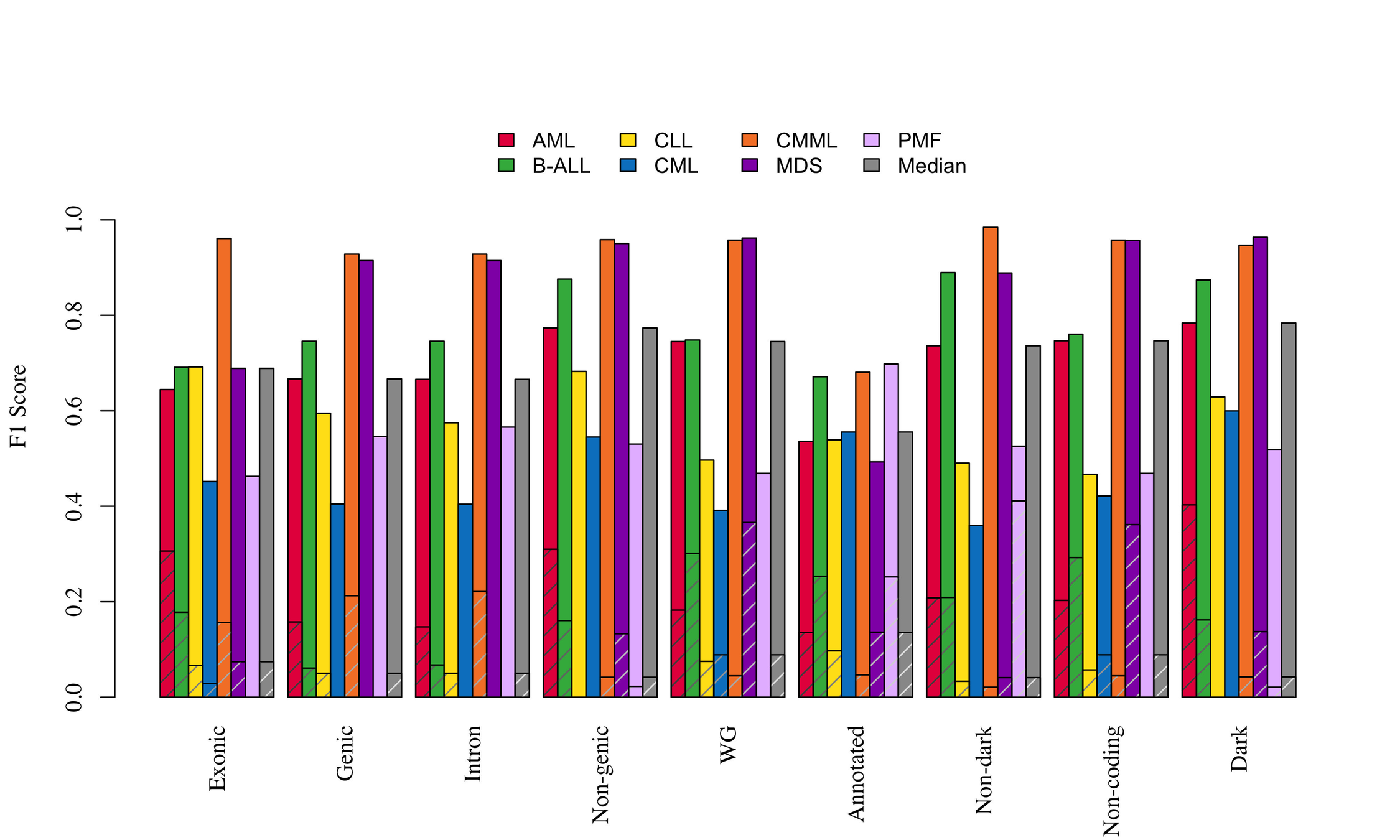

Supplement: S7 Fig — ReVeaL scores on disease-label permutations, used as negative controls, are shown in overlaid hatched bars. (TIF) [file pcbi.1007332.s015.tif]

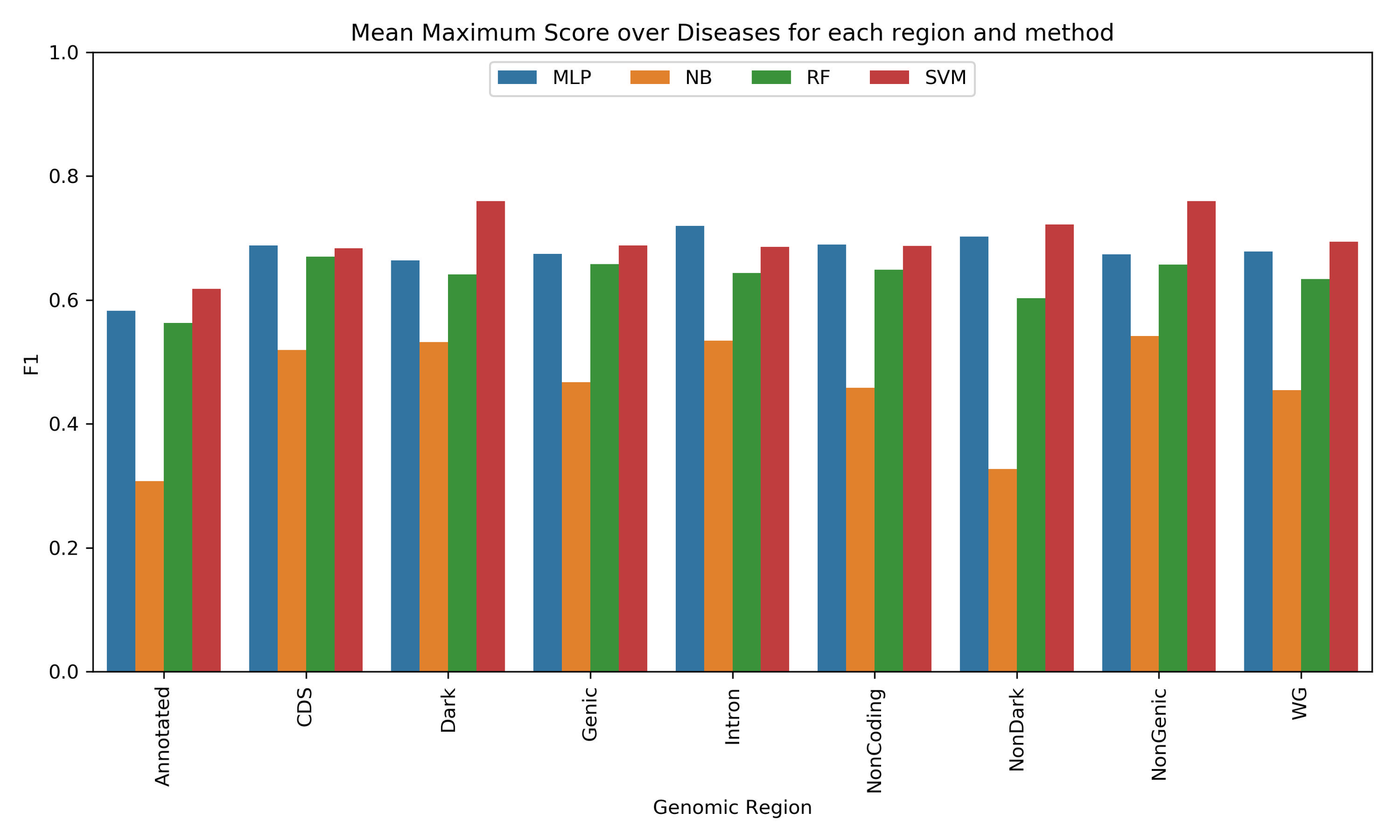

Supplement: S8 Fig — Each bar represents the mean over the set of maximum F1 scores achieved by any algorithm for a given disease. (TIF) [file pcbi.1007332.s016.tif]

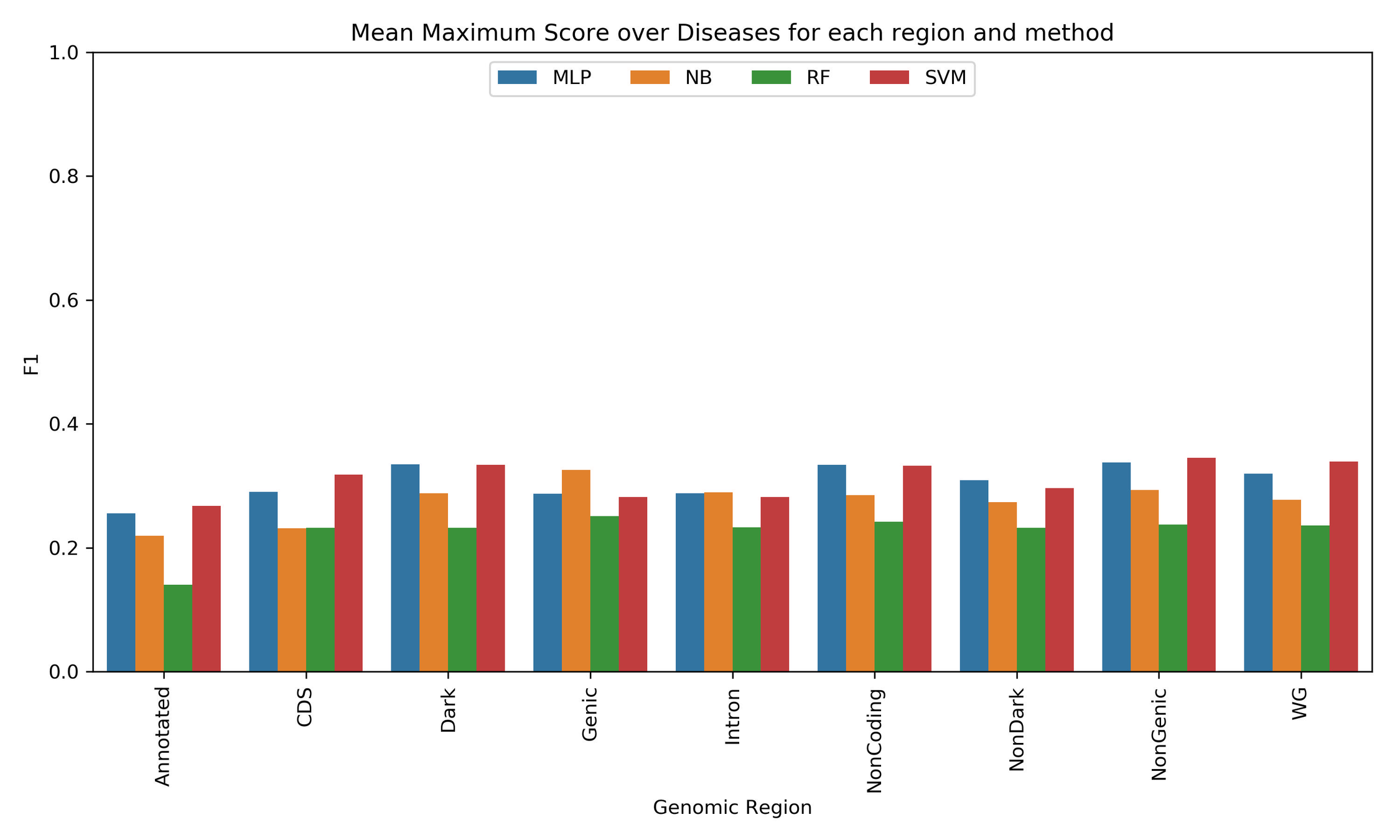

Supplement: S9 Fig — Each bar represents the mean over the set of maximum F1 scores achieved by any algorithm for a given disease. (TIF) [file pcbi.1007332.s017.tif]
